# Resistance to Thyroid Hormone Beta: A Focused Review

**DOI:** 10.3389/fendo.2021.656551

**Published:** 2021-03-31

**Authors:** Theodora Pappa, Samuel Refetoff

**Affiliations:** ^1^ Division of Endocrinology, Diabetes and Hypertension, Brigham and Women’s Hospital, Boston, MA, United States; ^2^ Departments of Medicine, Pediatrics and Committee on Genetics, The University of Chicago, Chicago, IL, United States

**Keywords:** resistance to thyroid hormone, thyroid hormone receptor, variant of unknown significance, autoimmune thyroid disease, thyroid dysgenesis, epigenetic effect

## Abstract

Resistance to thyroid hormone (RTH) is a clinical syndrome defined by impaired sensitivity to thyroid hormone (TH) and its more common form is caused by mutations in the *thyroid hormone receptor beta (THRB)* gene, termed RTHβ. The characteristic biochemical profile is that of elevated serum TH levels in absence of thyrotropin suppression. Although most individuals are considered clinically euthyroid, there is variability in phenotypic manifestation among individuals harboring different *THRB* mutations and among tissue types in the same individual due in part to differential expression of the mutant TRβ protein. As a result, management is tailored to the specific symptoms of TH excess or deprivation encountered in the affected individual as currently there is no available therapy to fully correct the TRβ defect. This focused review aims to provide a concise update on RTHβ, discuss less well recognized associations with other thyroid disorders, such as thyroid dysgenesis and autoimmune thyroid disease, and summarize existing evidence and controversies regarding the phenotypic variability of the syndrome. Review of management addresses goiter, attention deficit disorder and “foggy brain”. Lastly, this work covers emerging areas of interest, such as the relevance of variants of unknown significance and novel data on the epigenetic effect resulting from intrauterine exposure to high TH levels and its transgenerational inheritance.

## Introduction

The term resistance to thyroid hormones (RTH) refers to the clinical syndrome of reduced sensitivity to thyroid hormones (TH) first described in 1967 (1) and until recently it was synonymous with mutations in the *thyroid hormone receptor beta (THRB)* gene. In the past decade, mutations in the *THRA* gene, as well as genetic defects involving TH cell transport and metabolism were added to those of defects of TH action, broadening our understanding of impaired TH sensitivity (2–4).

This mini-review is dedicated to RTH due to mutations in *THRB* gene producing RTHβ, having as a signature elevated serum free iodothyronines levels but non-suppressed thyrotropin (TSH) in the absence of other conditions that may produce some of the characteristic test abnormalities. It focuses on emerging concepts, unusual associations and controversies involving diagnosis and management, while providing a succinct overview of RTHβ covered in most medicine and specialty textbooks (5, 6).

## Overview of RTHβ

As most neonatal screening programs are based on TSH measured in dry blood spots, the precise incidence of RTHβ is unknown. Surveys of 80,884 and 74,992 newborns using TSH and T_4_ measurements identified 2 and 4 infants with *THRB* gene mutations indicating a prevalence of 1 in 40,000 and 1 in 19,000 live births respectively (7, 8). Frequency among sexes is equal, whereas prevalence may vary somewhat among ethnic groups. The inheritance of RTHβ is typically autosomal dominant. This is explained by the formation of dimers between the mutant and normal (wild-type; WT) TH receptor (TR) interfering with the function of the WT TRβ. Since the first description of a *THRB* gene missense mutation causing RTHβ (9), 236 different mutations in 805 families have been identified. They are located in the functional areas of the ligand (T_3_)-binding domain and adjacent hinge region (10). In 14% of individuals manifesting the RTHβ phenotype no *THRB* mutations were identified. Rarely familial, they may be caused by mosaicism (11), whereas it has been postulated that mutations in enhancers, repressors or cofactors may be responsible for this subgroup of RTHβ (12).

The distinctive biochemical feature of RTHβ is high serum free iodothyronine levels (principally free T_4_) with normal or high TSH concentration. This discrepant correlation has brought the term “inappropriate TSH secretion”. Its wide use is deplorable as in fact the degree of TSH secretion is appropriate for the reduced sensitivity of the hypothalamic-pituitary axis to TH. Individuals with RTHβ maintain a nearly euthyroid state compensated by the high TH level in concert with the tissue expression level of the mutant receptor. Thus, features of TH deficiency and excess may co-exist, producing sinus tachycardia in the heart expressing mainly the WT TRα and goiter by TSH stimulation, as the pituitary expresses mainly TRβ including the mutant form. Visual disorders may also be present due to retinal photoreceptor dysfunction (13). Serum TSH determination remains the most sensitive test to determine reduced sensitivity to TH. In contrast, serum markers of TH action on peripheral tissues, such as cholesterol, creatine kinase, alkaline phosphatase, osteocalcin and sex hormone-binding globulin are less reliable, unless they are measured before and after administration of T_3_ (14).

After excluding assay interference as a cause of discrepant thyroid function tests (15), the principal other condition to be considered in the differential diagnosis of RTHβ is TSH secreting pituitary adenoma (TSH-oma), particularly in the absence of family history. Thus, testing of first-degree relatives is helpful and cost effective. Characteristics of a TSH-oma include failure to suppress TSH after the administration of supra-physiologic doses of T_3_, failure to normally stimulate TSH with TSH releasing hormone (TRH) (although exceptions of TSH-omas with TSH response to TRH have been reported), elevated sex hormone binding globulin levels and increased ratio of pituitary α glycoprotein relative to TSH (16). Co-secretion of growth hormone and prolactin and abnormal pituitary imaging on computerized tomography or magnetic resonance imaging are important diagnostic findings. However, incidental pituitary lesions may be found in up to 24% of patients with RTHβ (15), thus increasing the complexity in differential diagnosis and the value of hormonal investigation and dynamic testing. Conditions that increase the serum iodothyronine levels in the absence of thyrotoxicosis must be considered, including familial dysalbuminemic hyperthyroxinemia (FDH). In a recent study of Khoo et al., the presence of the albumin mutation R218H in FDH interfered with the measurements of free T_4_ and T_3_ by automated immunometric assays leading to misdiagnosis of FDH as RTHβ or TSH secreting tumor (17). The diagnosis of RTHβ becomes quite challenging in the presence of concomitant thyroid pathology, a subject addressed in greater detail below. Caution should be exercised in the reduction of TH levels with antithyroid medication and ablative therapies (radioactive iodine or surgery) as it leads to difficulty in the subsequent treatment of hypothyroidism.

## Combined RTHβ and Thyroid Dysgenesis

The diagnosis of RTHβ is challenging and its management complicated when it co-exists with other disorders, such as congenital hypothyroidism (CH) and thyroid dysgenesis. Children with RTHβ commonly have short stature, goiter and learning difficulties (14) and in association with CH will present high serum TSH and may exhibit hypothyroid symptoms when treated with standard levothyroxine doses. Five reports of RTHβ with CH due to ectopic thyroid tissue have been reported (18–22). Of note, the case reported by Guo et al., had a lingual thyroid with a typical RTHβ phenotype but no detectable mutations in the *THRB* gene (21).

Persistent serum TSH elevation is frequently encountered during the early treatment of CH despite reaching serum T_4_ level in the upper limit of normal. This has been attributed to a delayed maturation of the T_4_ mediated feedback control of TSH (23). Defining the cause of persistent TSH elevation and addressing it appropriately is of paramount importance, as undertreatment may adversely impact growth and mental development. When non-compliance and suboptimal treatment are excluded by measurement of serum T_4_ and T_3_, suspicion for co-existence of RTHβ should be raised and, when confirmed, treatment with supraphysiologic doses of levothyroxine aims to bring the serum TSH to near normal while following growth, bone maturation and cognitive development. When RTHβ and ectopic thyroid tissue co-exist, another reason to aim at TSH suppression is to prevent thyroid tissue expansion in anatomic locations, such as the base of the tongue, that may cause dysphonia and hemoptysis.

## Autoimmune Thyroid Disease and RTHβ

Autoimmune thyroid disease (AITD) is a common thyroid condition affecting the general population and its coexistence with RTHβ has been considered incidental (24, 25). However, in a study of 330 individuals with RTHβ and 92 unaffected first-degree relatives, subjects with RTHβ had an over 2-fold higher frequency of positive thyroid auto-antibodies (26), suggesting that this association is not coincidental. A proposed pathophysiologic mechanism by the group of Gavin et al. invoked chronic stimulation of intrathyroidal lymphocytes by elevated TSH in RTHβ leading to pro-inflammatory cytokine production and thyrocyte destruction (27). Yet, in the study of Barkoff et al., the prevalence of AITD by age group was not influenced by the TRβ genotype which argues against high TSH being the cause of AITD (26).

Previous studies have shown that TH activates the immune system by acting on thymic epithelial cells and by direct effect on neutrophils, natural killer cells, macrophages and dendritic cells (28, 29). TH augments dendritic cell maturation and induces pro-inflammatory and cytotoxic responses. Given that dendritic cells are involved in the pathogenesis of AITD (30, 31), this might be a pathway mediating the association between RTHβ and AITD.

## Variability in RTHβ Manifestation

RTHβ manifestations can be variable in tissue expression and in severity. The terms “generalized”, “isolated pituitary” and “peripheral tissue” resistance have been used to describe different clinical manifestations of RTHβ suggesting tissue variability in the resistance to TH. The term generalized resistance to TH (GRTH) was applied to most patients with RTHβ that appear to maintain a euthyroid state whereas pituitary resistance to TH (PRTH) referred to patients with RTHβ that have symptoms of thyroid excess in peripheral tissues or demonstrate changes in peripheral tissue markers compatible to TH action without significant suppression of TSH (32). A single patient with presumed isolated peripheral RTH (PRTH) was reported, in whom administration of high dose of liothyronine (L-T_3_) suppressed serum TSH but elicited no clinical signs of TH excess (33). Subsequently shown not to have a *THRB* gene mutation, this case likely represents acquired reduced sensitivity to TH through deiodinase-3 induced hormone inactivation. The clinical spectrum in RTHβ is quite broad and overlapping, even among carriers of the same *THRB* mutation and within the same family, suggesting that the classifications of generalized and pituitary RTHβ are rather semantics to describe a varying range of clinical signs and symptoms resulting from altered sensitivity to TH (34–36).

In some instances, the variability in the severity of the resistance to TH is readily explained on the basis of the character and position of the genetic defect. Homozygous *THRB* mutations are clinically more severe as they lack a WT TRβ and they interfere with the function of the WT TRα through heterodimerization (37, 38). Frame-shift mutations, producing a nonsense extension of the TRβ carboxyl terminus, interfere not only with ligand binding but also with interaction of the cofactors (39). Similarly, mutations with near normal ligand-biding can interfere with function through impaired binding to DNA (R243Q/W) (40, 41) and others (L454V and R383H) have altered binding to coactivators or corepressors (32, 42, 43) leading as in the case of R429Q (44) to more prominent suppression of TSH through predominant effect on genes negatively regulated by TH. Alberobello et al. (45) showed that when a single nucleotide polymorphism located in an intronic enhancer was associated with R338W, it produced pituitary specific over-expression of the mutant TRβ2 receptor illustrating the role of regulatory regions in tissue specific manifestation of RTHβ.

Differences in the level of expression of the mutant *THRB* allele relative to the WT in germline transmitted RTHβ have been shown in fibroblasts (46), but this was not found in another study (47). However, variable tissue expression of a mutant TRβ does occur in *de-novo* mutations resulting in mosaicism (11). The latter can also explain the failure to identify a *THRB* gene mutation in individuals with classical presentation of RTHβ when the only DNA source was circulating leukocytes. Finally, dramatic differences in phenotype observed among members of a family with the same *THRB* gene mutation have remained unexplained despite extensive genetic *in vivo* and *in vitro* functional studies (48).

## Current and Future Treatment Approaches

No specific therapy to fully correct the TRβ defect is currently available. Based on the mechanism producing the defect, it is clear that developing mutation-specific ligands would abrogate the dominant negative effect of the mutant TRβs, allowing the WT TRβ to elicit T_3_ mediated thyroid hormone action. In 2005, the laboratory of the chemist John Kho synthesized TH analogues able to abrogate the dominant negative effect of the TRβ mutants R2320C, R230H and R316H when tested *in vitro* (49). More recently Yao et al. (50) showed that roxadustat, a drug used to treat anemia of renal failure, had 3- to 5-fold higher binding to the TRβ mutants V264D, H435L and R438H than T_3_. However, none of these agonists have been tested *in vivo*. Similarly, the development of cell and tissue-specific TH antagonists could reduce the cardiotoxic effects of high serum TH levels acting on the WT TRα predominantly expressed in the heart. Therefore, as of this writing, management of TRβ is tailored to the individuals’ symptoms resulting either from tissue TH excess or deprivation. Goiter, hyperactivity and mental “clouding” are clinical features that benefit from judicious treatment with L-T_3_ without inducing side effects from TH excess.

Goiter is frequently observed in individuals with RTHβ but is usually of little consequence. However, in the occasion of larger symptomatic goiter, a surgical approach is usually ineffective, as goiter tends to re-occur. Therefore, it is logical to target TSH suppression to inhibit thyroid gland growth (51). An approach of administering supraphysiologic doses of T_3_ every other day (250 µg in the case of TRβ R243Q) was successful in drastically reducing goiter size in a young patient without inducing thyrotoxic symptoms, as serum T_3_ rapidly declined reaching levels lower than baseline before the ingestion of the next L-T_3_ dose (52). The rationale is to deliver a large dose of the short lived L-T_3_ to achieve very high peak serum level suppressing the TSH below 0.1 mIU/L to inhibit thyrocyte growth without sustaining elevated TH levels long enough to cause thyrotoxic symptoms (52). Thyroid nodules are quite prevalent in the general population and thus may occasionally co-exist with RTHβ. Although the majority of thyroid nodules are benign and do not require surgical management, there are few reported cases of papillary thyroid carcinoma in patients with RTHβ. In these cases, thyroidectomy and radioactive iodine ablation to prevent disease recurrence result in lifelong levothyroxine replacement therapy, and in RTHβ persistently high serum TSH. Although the outcomes in the reported cases were fortunately not unfavorable, levothyroxine therapy is challenging and supraphysiologic doses are often needed to maintain serum TSH in lowest tolerable level (53). Alternative options to consider include 3,3,5-triiodothyroacetic acid (Triac), a thyroid hormone analogue with thyromimetic effects on pituitary and liver tissue that may be used to suppress TSH, combination of levothyroxine with beta-blocker to alleviate tachycardia along with calcium and vitamin D supplementation to prevent bone loss acceleration. Lastly, surveillance strategy may be considered for occult, micro-papillary thyroid carcinomas with low potential for aggressive progression.

Attention deficit disorder (ADHD), reported in 48-83% of individuals with RTHβ, is treated using conventional drugs. When such medications are ineffective, treatment with L-T_3_ was found beneficial in reducing impulsivity in 5 of 8 and hyperactivity in 4 of 7 individuals with RTHβ and ADHD but not in individuals with ADHD only (54). Every-other-day L-T_3_ therapy was also effective to improve the insomnia and hyperactivity in a young child with severe RTHβ phenotype intolerant to daily L-T_4_ therapy (55).

The success of treatment with intermittent high dose L-T_3_ in improving brain function seems to be linked to the reduction of serum T_4_, a hormone more readily available to the brain which expresses predominantly TRα, providing a thyrotoxic local environment. This would be the rationale to consider block-and-replace strategy, proposed by Dr. Alexandra Dumitrescu, and used by the senior author to ameliorate “foggy brain” and anxiety occasionally reported by RTHβ patients, whereas beta blockade may be employed to help with tachycardia.

Lastly, Triac with higher affinity than T_3_ for several TRβ mutants may be used to diminish the dominant negative effect of a TRβ mutation. Further, though its short half-life, Triac can effectively reduce TSH with lesser thyromimetic effect on peripheral tissues (56). Triac therapy has been used in few RTHβ cases and was found beneficial in partially alleviating thyrotoxic symptoms including tachycardia, excessive perspiration, attention deficit disorders, as well as goiter. This was the case in patients harboring mutations in the ligand binding domain (residues 310-353 and 429-460), whereas two cases with mutations in the hinge region were refractory to Triac (56, 57). Notably, in a pediatric case of a homozygous *R243Q* mutation with features of thyrotoxicosis and early dilated cardiomyopathy, combination of Triac with methimazole resulted in reduction of thyroid hormones levels and normal TSH accompanied by lower basal metabolic rate and improved growth and cardiac function (58).

**Figure 1:**
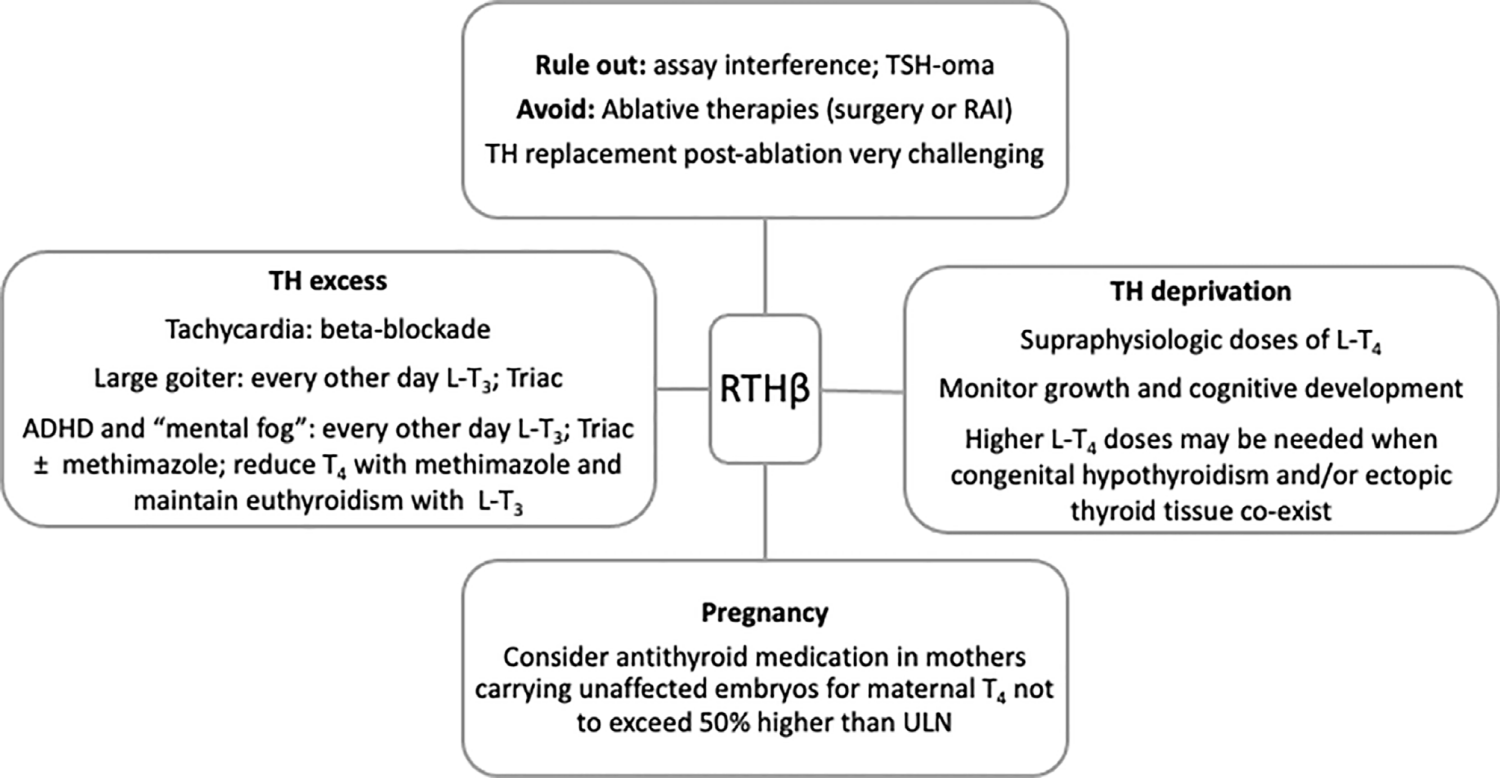
Summary of recommendations to guide clinical management of RTHβ. RAI, radioactive iodine; ADHD, attention deficit and hyperactivity disorder; TH, thyroid hormones; ULN, upper limit of normal.

A summary of recommendations to guide clinical management of subjects with RTHβ is presented in **Figure 1**.

## The Impact of *TRβ* Variants of Unknown Significance

The development of next generation sequencing (NGS) and its increased availability in clinical practice leads to identification of variants of unknown significance (VUS). These include variants of the *THRB* gene not previously reported to be associated with RTHβ. The interpretation of such genetic reports, particularly missense mutations, poses a problem to the practicing physicians; how to explain the findings to the patient and how to proceed with future care. *In vitro* functional analyses of VUS are not commercially available and results cannot be deduced with certainty even when they are.

*THRB* gene mutations are clustered in three regions of the ligand-binding domain of the TRβ. Yet a major region devoid of mutation (“cold area”) contains CG-dinucleotides which are mutagenic hot spots. Artificial mutations created in these CGs produced TRβs weak in dominant negative effect explaining the failure to identify mutations in this region of the receptor (59). This is explained by the fact that the same region is included in the dimerization domain. This region originally encompassed codons 348-437. Later, with the identification of *THRB* gene mutations causing RTHβ, the “cold region” was narrowed down to encompass codons 384-425 (32, 60). Within this region, 12 variants (P384L, G385R, L386V, E390D, R391K, D397G, S398G, N408S, H413D, V414M, K420R, and V425L) were reported in the gnomAD database without information regarding clinical phenotype (61). Although most variants are considered benign based on *in silico* prediction algorithms, conflicting predictions were made for the P384L, D397G and K420R variants and the G385R variant was considered damaging (62). Recently, a 48 year-old patient with AITD, treated with levothyroxine, was found to have high free T_4_ with non-suppressed TSH. A mutant TRβ G385E was identified and reported as VUS. Family screening uncovered the same mutation in relatives with normal thyroid function, suggesting that this mutation may not be responsible for the abnormal thyroid pattern (63). Similarly, the G339S variant was identified in a family with AITD after an individual was misdiagnosed with RTHβ, but the same variant was then found in several family members with normal thyroid function, making it unlikely for the G339S variant to be causally related to a RTHβ phenotype (24).

The above paradigms illustrate that *in silico* prediction algorithms may not always be reliable when studying the functional relevance of VUS. Genotype-phenotype co-segregation among family members is useful in characterizing the functional impact of *THRB* mutations. Computational resources that factor in protein specific functional domains may have some predictive functional relevance of VUS but should not be the basis guiding clinical decision making.

## Epigenetic Effect of RTHβ and its Transgenerational Inheritance

The first body of evidence on fertility and pregnancy outcome in RTHβ came from studies in a large Azorean kindred harboring the R243Q mutation. Fertility was not affected and, contrary to women with thyrotoxicosis, RTHβ did not produce an increase in premature labor, stillbirth or pre-eclampsia, jn agreement with the women’s euthyroid state despite elevated TH levels (64). However, a significantly higher rate of early miscarriages was observed in women with RTHβ compared to spouses of males with RTHβ or unaffected first-degree relatives independent of maternal age and parity. Furthermore, a tendency was seen for these women to miscarry unaffected fetuses rather than fetuses with RTHβ, suggesting that the miscarriages occurred due to fetal exposure to incongruent high TH levels. In addition, unaffected newborns of mothers with RTHβ had significantly lower birth weight and suppressed TSH at birth compared to offspring of unaffected mothers, arguing that they were exposed in a hypercatabolic intrauterine environment of high TH concentration, whereas infants with RTHβ were protected from the toxic effect of TH excess. Of note, when women with RTHβ carrying unaffected fetuses were given antithyroid medication to avoid free T_4_ levels 20% higher than the upper limit of normal, the birth weight and TSH levels at birth of their offspring was similar to infants with RTHβ (65).

**Figure 2:**
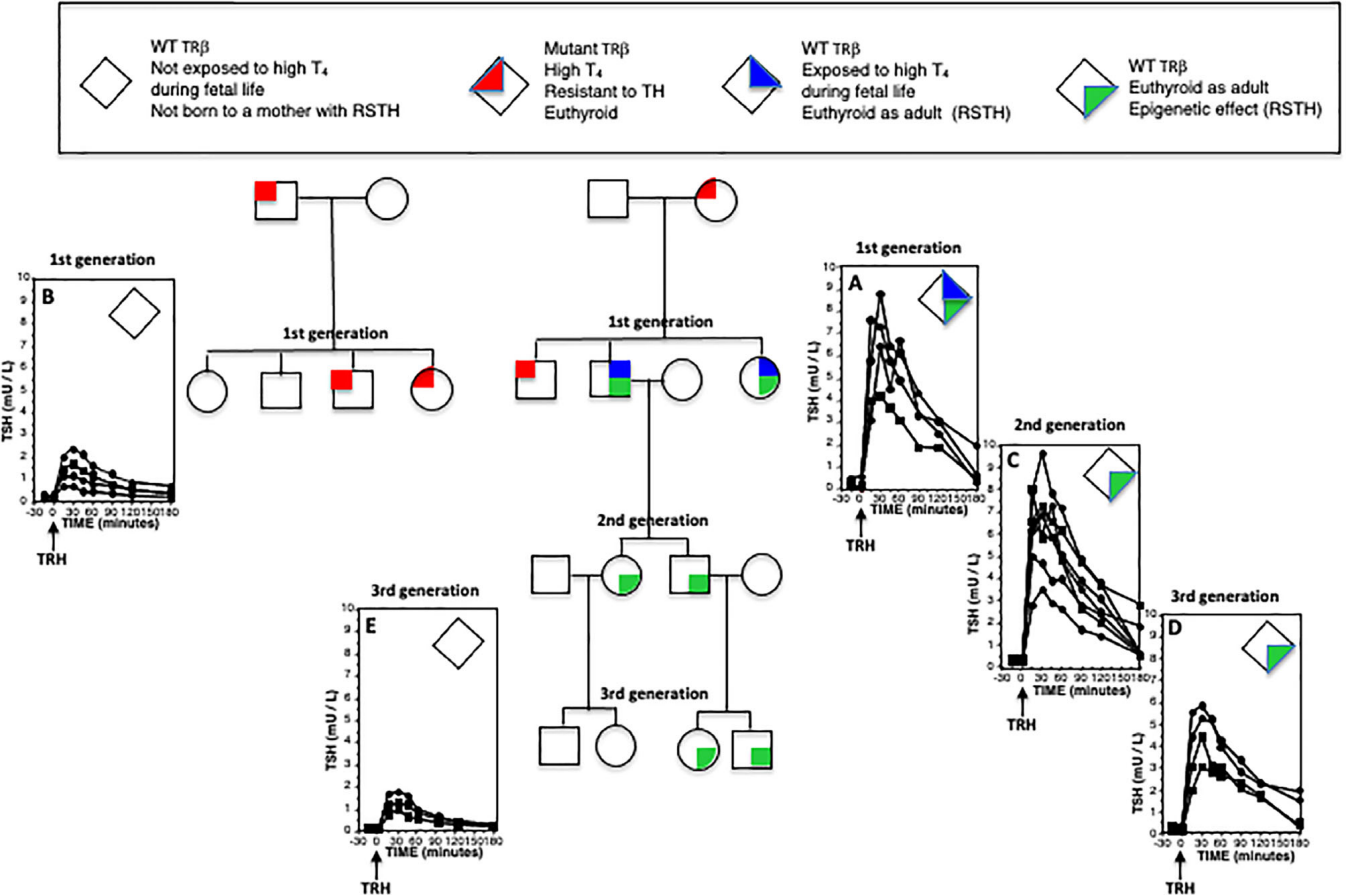
Epigenetic effect of RTHβ and its transgenerational inheritance across the male line. Individuals were given 25 µg L-T_3_ twice daily for three days. They were then injected intravenously with 200 µg of thyrotropin releasing hormone (TRH) and samples of blood were obtained at the indicated times for the measurement of serum TSH. Plotted on the graphs are results from female (circles) and males (squares). Increased peak responses in **(A, C, D)**, as compared to those of **(B, E)** indicate reduced sensitivity to thyroid hormone (RSTH). While this epigenetic effect of exposure to high TH levels during fetal life **(A)** is transmitted to both sexes, it is inherited along male line only **(C, D)** but not the female line **(E)**.

In a subsequent study, the long-term effect of intrauterine exposure to high TH levels was examined in WT members of the Azorean kindred. Specifically, the study involved unaffected offspring of mothers with RTHβ and offspring of unaffected mothers, whose fathers had RTHβ, as well as mice mimicking the human phenotype. Unaffected humans and WT mice born to mothers with RTHβ and exposed to high TH levels *in utero* developed reduced central sensitivity to thyroid hormone (RSTH), that persisted during adulthood (66) (**Figure 2**). Increased expression of deiodinase 3, the enzyme that inactivates TH, was found in the pituitaries of the WT mice born to dams with RTHβ (66). This effect was found to be transmitted by male descendants but not in female with likewise RSTH (67). Although the exact mechanism of this transgenerational epigenetic inheritance is not fully characterized, it is thought to involve possible modulation of the imprinted deiodinase 3 gene that regulates local TH availability at a tissue specific level. It remains unclear whether prolonged exposure to high TH levels could have similar implications in adult life. This deserves further investigation as such a finding would have implications in the management of larger populations, such as individuals on long term TSH suppressive levothyroxine therapy for differentiated thyroid cancer.

## Discussion–Conclusions

The diagnosis of RTHβ is challenging and the main condition in the differential diagnosis is TSH-oma. Diagnosis and management of RTHβ are more challenging when other thyroid disorders co-exist, such as CH and ectopic thyroid tissue. More recently, an association has been described between RTHβ and AITD. Although the causal relation remains unclear, proposed pathophysiologic mechanisms include TSH or TH induced stimulation of pro-inflammatory and cytotoxic responses. The observed variability in clinical manifestation of RTHβ can be explained by the type of genetic defect, e.g. homo- *vs* hetero-zygosity, frameshift *vs* insertion/deletion, mutations with predominantly TRβ2 mediated action, mosaicism, and the tissue specific variability in TRβ expression, e.g. heart and brain *vs* pituitary and liver. Management is tailored to control symptoms arising from tissue specific excess or lack of TH. In small case series treatment with every-other-day L-T_3_ was beneficial in improvement of goiter and ADHD symptoms. When RTHβ co-exists with CH, supraphysiologic doses of L-T_4_ are needed to achieve normal bone and cognitive development. The advances in NGS have led to increasing frequency of VUS identification, where there may be limited data on their functional relevance beyond *in silico* prediction models. Caution should be exercised as to not guide clinical decision making based on computational resources and utilize information from genotype-phenotype co-segregation in family members. Transgenerational studies in humans and mice provide evidence of an epigenetic effect induced by RTHβ, by *in utero* exposure of WT fetuses to high TH concentration. The resulting reduced sensitivity to TH shows transgenerational inheritance across the male but not the female line and is thought to be mediated *via* modulation of deiodinase 3, that regulates local TH availability.

The advances in our knowledge on RTHβ raise novel questions about TH action outside the hypothalamus-pituitary-thyroid axis and the emerging concepts on epigenetic effect of RTHβ need to be explored further, as they may have implications in larger populations, such as patients with thyroid cancer on long term TSH suppression therapy with TH.

## Author Contributions

TP and SR designed and wrote this manuscript and both conceptually contributed to this work. All authors contributed to the article and approved the submitted version.

## Funding

This work was supported in part by grant DK15070 from the National Institutes of Health. The content is solely the responsibility of the authors and does not necessarily represent the official views of the National Institute of Diabetes and Digestive and Kidney Diseases or the National Institutes of Health. TP is supported by the NIH T32 grant 5T32HL007609-33.

## Conflict of Interest

The authors declare that the research was conducted in the absence of any commercial or financial relationships that could be construed as a potential conflict of interest.
